# VPA: an R tool for analyzing sequencing variants with user-specified frequency pattern

**DOI:** 10.1186/1756-0500-5-31

**Published:** 2012-01-14

**Authors:** Qiang Hu, Dan Wang, Li Yan, Hua Zhao, Song Liu

**Affiliations:** 1 Department of Biostatistics, Roswell Park Cancer Institute, Buffalo, New York 14263, USA; 2 Department of Cancer Prevention and Control, Roswell Park Cancer Institute, Buffalo, New York 14263, USA

## Abstract

**Background:**

The massive amounts of genetic variant generated by the next generation sequencing systems demand the development of effective computational tools for variant prioritization.

**Findings:**

VPA (Variant Pattern Analyzer) is an R tool for prioritizing variants with specified frequency pattern from multiple study subjects in next-generation sequencing study. The tool starts from individual files of variant and sequence calls and extract variants with user-specified frequency pattern across the study subjects of interest. Several position level quality criteria can be incorporated into the variant extraction. It can be used in studies with matched pair design as well as studies with multiple groups of subjects.

**Conclusions:**

VPA can be used as an automatic pipeline to prioritize variants for further functional exploration and hypothesis generation. The package is implemented in the R language and is freely available from http://vpa.r-forge.r-project.org.

## Background

The widespread availability of next-generation sequencing (NGS) technology provides an unprecedented opportunity to screen the full spectrum of disease-causing genetic variations [1]. Concomitant with the rapid development of sequencing technology, many analytic methods have been developed to convert the raw sequencing data into variant and sequence calls [2]. In NGS study of human subjects, the average number of sequence and variant calls often ranges from ~26 million positions and ~20 000 variants per exome subject [3], to ~2.6 billion positions and ~3 500 000 variants per genome subject [4,5]. The high-volume nature of sequence and variant calls generated from NGS study thus calls for the development of computational tools to prioritize them for downstream analysis. From a statistical point of view, effective variant prioritization will reduce the burden of multiple comparisons and increase the power to catch casual variants associated with the phenotypes of interest.

Several tools exist to annotate the functional impact of voluminous variants detected from high-throughput sequencing studies [6-11]. One common strategy of them is to compare the detected variants to the precompiled annotation libraries (e.g., Emsembl gene annotation) for annotation and/or catalogs of known polymorphisms (e.g., dbSNP database) for filtering. However, in study with more than one subjects being sequenced, it is additionally crucial to prioritize variants with desired frequency pattern across the study subjects of interest. The frequency pattern can range from somatic type of variant (i.e., variants occurring in case but not matched control) to variants with user-specified distinct frequencies in different subject groups characterized by different phenotypes. The allele frequency of genetic variants has been reported to be associated with the likelihood of disease-causing potentials [12]. Here, we present VPA (Variant Pattern Analyzer), a specialized R package that enables users to simultaneously parse the variant and sequence call files of multiple subjects to extract variants with user-specified frequency pattern. The tool allows incorporating several position-level quality criteria recorded in the variant and sequence call files into variant extraction. The package is flexible for users to tailor within R (> = 2.12.1 for Unix-like operation system; > = 2.14.0 for Windows operation system) statistical computing and graphics environment for their specific needs.

## Implementation

### Input files

For each genomic position of interest, VPA will check for variant call information for the subject with variant called at this position, and it can also check for sequence call information for the subjects without variant called at the same position. The option of scrutinizing sequence call information for the subjects without variant called at the position of interest, instead of simply intersecting variant call information of all subjects, is to exclude positions with incomplete sequencing (i.e., low sequencing depth) in certain subjects from variant frequency pattern analysis. Therefore, the input for VPA consists basically of two types of files for each subject, containing position-level information of variant calls (required) and sequence calls (optional). The supported format of input files is Variant Call Format (VCF) [13], which can be generated from state-of-the-art raw data analysis tools [14,15]. For computational efficiency, the high-volume data in tab-delimited VCF formats can be compressed by bgzip program and retrieved through tabix program. Both bgzip and tabix are available as part of the open source SAMtools package [14].

### Usages

In this section we will briefly introduce VPA's basic commands to parse the variant data of multiple sequencing subjects to extract variants with user-specified frequency pattern and quality criteria. In an exemplary application of three-group design (e.g., group with aggressive phenotype, group with benign phenotype, and group with normal phenotype) with 5 subjects per group, our goal is to extract variants recurrent in aggressive phenotype group (i.e., frequency ≥ 0.4), not recurrent in benign phenotype group (i.e., frequency ≤ 0.2), and not observed in normal phenotype group at all (i.e., frequency = 0.0). To achieve that, the **LoadFiltering **function will be first used to load the data and perform position-level quality filtering as follows:

• **Varfit < - LoadFiltering(file = "index.txt", filtering = TRUE)**

The **index.txt **file contains group status and VCF file location of each subject. For each genomic position of interest, VPA will retrieve variant call information from the subject(s) with variant called at this position. If the optional sequence call files are provided, it will make use of tabix function [14] to retrieve sequence call information from the subject(s) without variant called at the same position. It will filter out the variant positions which don't reach the user-specified quality criteria from further frequency analysis. The current position-level quality criteria implemented in VPA includes minimum and maximum sequencing depth, number and percent of reads containing variant allele, phred-scaled variant quality, phred-scaled genotype quality, and normalized, phred-scaled likelihoods for each genotypes. These quality criteria can be set in the optional argument of **LoadFiltering **function.

Once an object for the list of high-quality genomic position of variant call is obtained, the Patterning function can be used to identify variants with frequency > = 0.4 in aggressive group, < = 0.2 in benign group and = 0 at normal group as follows:

• **Pattern < - cbind (Aggressive = c(0.4, 1.0), Benign = c(0.0, 0.2), Control = c(0.0, 0.0)))**

• **varpat < - Patterning (varfit, pattern)**

The resulting **varpat **object contains variants that fit the desired frequency pattern and their detailed frequency distribution. The former results can be exported by VPA to plain text files in VCF format for further annotation analysis. The latter results can be exported by VPA to standard excel report for data summary.

We have implemented multiple functions to analyze the frequency pattern at both variant and gene levels, as well as options (i.e., Fishers' exact test and Chi-square test) to assess the statistical significance of observed frequency difference. The VPA package also includes functions to filter variants against known variants dataset such as dbSNP, 1000 genome project data and custom VCF data file. A complete list of functions in the package VPA is shown in Table 1. A complete description of package functionality and executable examples can be found in the package vignettes and manual of VPA.

**Table 1:** A complete description of package functionality and executable examples can be found in the vignette and manual of VPA

Function	Description
LoadFiltering	Load data from study subjects and perform position-level quality filtering

Patterning	Prioritize sequencing variants in user-specified frequency pattern

read.vcf	Read data file in VCF format

write.vcf	Write a VCF format object to a file

filtervcf	Filter sequencing variants with user-specified quality criteria

filterpos	Filter sequencing variants against known SNP datasets

Pos2Gene	Annotate sequencing variants into genes

gefreq	Frequency analysis at the gene level (for aggregates of variants)

subvcf	Extract a subset of variants from a VCF format object

pos2seq	Retrieve information for position of interest from tabix indexed data file

vcfreq	Summarize the frequencies of sequencing variants

### Performance

We evaluated the computational efficiency of VPA using a human exome sequencing dataset with 5 case subjects and 5 control subjects. On average there are about 91, 000 variants and 24,000,000 sequence calls in each subject. The first step of the pipeline is to load all variant call data and performing position-level quality filtering. It took only 7.7 minutes to complete this step in a single Intel Xeon 2.27 GHz of CPU with 24 GB of memory. The optional sequence call checking will be much more time-consuming as it relies on tabix function to retrieve positions of interest from the huge list of sequence call. To achieve efficiency for this optional application, the **LoadFiltering **step has been implemented in both sequential and parallel modes. In the sequential mode, it took about 4.4 hours in a single Intel Xeon 2.27 GHz of CPU with 24 GB of memory. However, it only took about 1 hour in parallel mode using a cluster server of 10 CPUs with similar setting. Once the quality filtering is done, the **Patterning **step costs much less computing time. It only took about 4 minutes to identify and extract variants with specified frequency pattern (e.g., recurrent in case group but not occurring in control group).

## Conclusions

The applications of high-throughput sequencing instruments in biomedical studies are generating huge volumes of sequence and variant call data. A number of public tools for variant prioritization utilizing up-to-date annotation resources have been proposed. The VPA tool allows the users to prioritize a list of sequencing variants with desired frequency pattern in various next-generation sequencing experiment designs. Written in open source R environment, it provides the flexibility for users to adopt, extend and customize the functionality for their specific needs. To our knowledge, at the time of writing there is no public R package available for this important utility. VPA is easy to use. It can be used either before or after annotating the variants using precompiled annotation databases. It can be used in individual studies as well as in situations where the sequencing data are collected from different studies.

We will continue extending the functionality of VPA package. For example, the currently supported format of input data is VCF file, which can be converted from raw BAM files using standard analytic tools like SAMtools or GATK. Plans of future improvement for VPA package will include new functions to retrieve relevant information from the BAM files. The current implementation of VPA package allows users to exclude positions with low sequencing depth from frequency pattern analysis. As methods to effectively impute the genotypes at low coverage are under active development [16], future development of VPA package will include functions to handle imputed genotypes with low sequencing depth. Furthermore, we will continue to extend the position-level quality criteria to filter out erroneous variants from frequency pattern analysis.

## Availability and requirements

**Project name: **Variant Pattern Analyzer

**Project home page: **http://vpa.r-forge.r-project.org

**Operating system(s): **Windows, Unix-like (Linux, Mac OSX)

**Programming language: **R (For Unix-like operation system, the version of R should be > = 2.12.1; For Windows operation system, the version of R should be > = 2.14.0)

**License: **GNU GPL

**Any restrictions to use by non-academics: **None

## Competing interests

The authors declare that they have no competing interests.

## Authors' contributions

QH and SL developed the software and drafted the manuscript. DW, LY and HZ provided discussion of ideas and assisted in preparing the manuscript. All authors read and approved the final manuscript.
